# The Hurst Exponent as an Indicator to Anticipate Agricultural Commodity Prices

**DOI:** 10.3390/e25040579

**Published:** 2023-03-28

**Authors:** Leticia Pérez-Sienes, Mar Grande, Juan Carlos Losada, Javier Borondo

**Affiliations:** 1 Grupo de Sistemas Complejos, ETS Ingeniería Agronómica, Alimentaria y de Biosistemas, 28040 Madrid, Spain; 2 AgrowingData, Navarro Rodrigo 2 AT, 04001 Almería, Spain; 3 Departamento de Gestión Empresarial, Universidad Pontificia de Comillas ICADE, Alberto Aguilera 23, 28015 Madrid, Spain

**Keywords:** efficient market, time series, agri-food, Hurst, market, prices, agriculture

## Abstract

Anticipating and understanding fluctuations in the agri-food market is very important in order to implement policies that can assure fair prices and food availability. In this paper, we contribute to the understanding of this market by exploring its efficiency and whether the local Hurst exponent can help to anticipate its trend or not. We have analyzed the time series of the price for different agri-commodities and classified each day into persistent, anti-persistent, or white-noise. Next, we have studied the probability and speed to mean reversion for several rolling windows. We found that in general mean reversion is more probable and occurs faster during anti-persistent periods. In contrast, for most of the rolling windows we could not find a significant effect of persistence in mean reversion. Hence, we conclude that the Hurst exponent can help to anticipate the future trend and range of the expected prices in this market.

## 1. Introduction

Financial markets are extremely complex systems with a large number of interacting units, and anticipating their evolution is far from straightforward. Thus, their study has attracted the attention of researchers over the past decades.

An active and relevant topic of discussion among researchers is whether or not the financial market prices display long memory properties. The importance of this question lies in its consequences for market theories and its predictability. The fact that a market presents a long time memory implies that prices do not follow a random walk, as there is autocorrelation, and they are therefore predictable. On the other hand, if there is no memory, the Efficient Market Hypothesis (EMH) [1,2] cannot be rejected. The EMH was introduced by Fama in 1970 and states that new information is immediately reflected in the asset prices and therefore show martingale behavior. According to this theory, price changes are not related to the historical behavior of price volatility, but represent a response to new information, and since this arrives randomly, the evolution of prices is unpredictable. In the current literature there are papers supporting both hypotheses. Several authors have shown evidence of markets that present long time memory [3,4,5,6,7,8,9] whereas other authors have found evidence supporting the EMH [10,11,12].

In nature we find several examples of physical systems that do present long time memory properties, such as radiation or rainfall. Thus, researchers from econophysics, inspired by the idea that the financial system may share the same properties, have also attempted to detect trends and patterns in financial time series that can help to anticipate its trend. This discipline is known as Technical Analysis [13,14].

Due to the high importance of long time memory for the predictability of time series, there is a clear need for a method that identifies the existence or not of this memory. Currently, the Hurst exponent (H) is the most widely used and accepted test to measure long-term memory properties [10,15]. H is a measure for long term memory and fractality of a time series that quantifies the degree of persistence of similar change patterns. This analysis was originally introduced by Hurst in 1951 to study the storage capacity of reservoirs in the Nile River, taking into account the cyclical trends of the flow, drought periods, and floods. This work was popularized and extended to other disciplines in the 1960s by Benoit Mandelbrot [16,17,18], who claimed that this methodology was superior to the autocorrelation, the variance analysis, and to the spectral analysis. Since then, several other methods of Hurst calculation have been developed. The best known ones are Rescaled Range [15], Detrended Fluctuation Analysis [19,20], wavelet transforms [21], and Generalized Hurst Exponent [22].

*H* ranges between 0 and 1, and provides information on whether the series presents long-term or not. If H=0.5, then each step is independent of the past values of the series. Thus, there is no memory and the series is equivalent to white noise. Under this setting, the time series is unpredictable and the EMH is fulfilled. When H≤0.5, the series is anti-persistent. In this scenario, the series is expected to display ‘mean-reversion’. This fact implies that increments are generally followed by a decrease, while drops are followed by an increment. Finally, when H≥0.5, the series is persistent. In a persistent regimen, the series is more likely to maintain the trend in a broader range than what is expected by pure random walk. Thus, a rise in the previous step will most likely be followed by another rise, while a fall will be followed by another fall.

Long-term memory is an important feature of market dynamics with implications for its predictability. As a consequence, the Hurst exponent has been widely applied to study the stock, currencies markets, and, more recently, to cryptocurrencies [23,24]. For instance, Di Matteo et al. [12] show how H serves as an index to classify mature and emergent markets. In the same line of research, Bianchi et al. [25], used the Hurst–Hölder exponents to detect periods of efficiency and inefficiency in stock markets [26]. Other researchers have analyzed how to use H to find the most profitable trading pairs [27], concluding that H performs better when compared with the classical methods. Despite the wide use of H in the stock market, there is still limited research on its applicability to the agri-commodities market [28,29,30]. In this paper we will study the agri-commodities market, and more particularly the evolution of prices for four horticultural products. Understanding this market is becoming increasingly important to make the agri-food industry sustainable [31,32], as price crises result in a waste of food.

The stock market and the agri-commodities market have some similarities, but at the same time also have some important differences. On the one hand, both of them represent a market that is driven by demand. Matia et al. [33,34] showed that the two markets share several properties, although they also found some differences. The cumulative distribution of returns can be adjusted to a power law for both markets. In addition, the returns for the stocks and commodities market exhibit a multifractal behavior. On the other hand, there are differences in the nature of these two markets. In contrast with the stock market, in agri-commodities markets, commodities represent a physical product that has to be stored and transported, and in some cases it is even a fresh and perishable product. Moreover, for agri-commodities we can expect slower changes and response to the demand, since the market is very conditioned by the supply of each product.

In this paper, we will explore the applicability of H to the agri-food market in order to anticipate the trend and range of the future price. In particular, we focus on fresh vegetables because price crises have a big impact on them, since they are perishable products that can not be stored. Thus, when co-ops fail to anticipate the price and can not market their production, it results in tons of wasted food. The effect of the long memory properties on the agri-commodities markets has still attracted little attention from researchers. Thus, there is a gap in the current scientific literature, which misses to fully understand the behavior of such markets. In Ref. [35] the authors analyze the auto-correlations and cross-correlations of the volatility time series for the Brazilian stock and commodity markets. They found auto-correlations in the commodity market, which in fact are stronger than that observed for the stock market. In another study—see Ref. [30]—the authors computed the Hurst exponent for several commodity price series, and found that most commodity prices are consistent with the underlying assumption of a geometric Brownian motion. We will contribute to understanding the dynamics and properties of the market by analyzing the evolution of H over time, and evaluating whether the value of H can provide useful information to anticipate the future trend of the price. In addition, we will compare the results obtained when computing H for different time windows.

The present paper is organized as follows. In Section 2, we will explain the methodology followed to compute the local Hurst exponent of the series and the mean reversion. Next, in Section 2.3, we describe our data. In Section 3, we expose our results. Finally, in Section 4, we present our conclusions and discuss the importance of our results.

## 2. Materials and Methods

### 2.1. Hurst Exponent

The Hurst exponent (H) is used in time series analysis and fractal analysis as a measure of the long-term memory of a time series. In other words, *H* measures how chaotic or unpredictable a time series is. In the literature, we can find several methods to calculate *H*, such as re-scaled Range (RS) [15], Detrended Fluctuation Analysis (DFA) [19,20], wavelet transforms [21], and Generalized Hurst Exponent (GHE) [22].

In this work, we use the GHE algorithm in order to measure the long-term memory of the price time series of different agri-commodities. This method is based on the scaling behavior of the statistic:

(1) $$K_{q}\left(\tau \right)=\frac{\langle |X\left(t+\tau \right)-X\left(t\right){|}^{q}\rangle }{\langle |{\left(X\left(t\right)\right)}^{q}|\rangle },$$

which is given by

(2) $$K_{q}\left(\tau \right)\propto \tau^{qH},$$

where τ is the time scale and can vary between 1 and $\tau_{max}$, *H* is the Hurst exponent, <·> denotes the sample average on time *t*, and *q* represents the order of the moment considered.

*H* is then calculated by taking logarithms in relation (2) for different values of τ. In this paper, we work with $\tau =2^{n}$$\left(n=0,1,\ldots ,log_{2}\left(N\right)-2\right)$, and q=1, as $H_{1}$ is the closest estimation to the classical Hurst exponent [12].

*H* ranges between 0 and 1, where H=0.5 means that there is no memory and the series is equivalent to white noise. When H≤0.5, the series is considered anti-persistent and is expected to display ‘mean-reversion’. Finally, when H≥0.5, the series is considered persistent and is more likely to maintain the trend in a broader range than what is expected by a pure random walk.

In order to prevent *H* from using future values of the time series, we calculate a local Hurst exponent with reference to a rolling window of 4, 8, 16, 32, and 52 weeks that ends the day of measurement. This method ensures that we use only past data to determine *H*.

Note that in order to compute H, we have coded the described method using Python.

### 2.2. Days to Mean Reversion

Mean reversion (MR), or reversion to the mean, is a theory used in finance that suggests that a measure of interest such as the price of a commodity or asset eventually reverts to its long-term average levels. Thus, this theory assumes that a variable that deviates far from its long-term trend will return, reverting to its average value. This concept has been used to define many investment strategies that seek to purchase or sell financial products whose recent market price differs greatly from their historical average [36].

In this work we are going to test whether this reversion is more likely to occur during anti-persistent periods rather than during persistent periods in the price time series. Thus, for each day *i*, we compute the number of days (d) that the price (p) of an agri-commodity lasts to revert to its average value (m) as

$$d=j^{*}-i$$

where $j^{*}$ is the minimum *j* that satisfies

$$\left\{\begin{array}{cc} p_{j}\leq m_{i}+\epsilon , & \;\mathrm{if}\;p_{i}\geq m_{i}\\ p_{i}\geq m_{i}-\epsilon , & \;\mathrm{if}\;p_{i}<m_{i} \end{array}\right.$$

with j≥i.

The average value (m) of each price time series have been calculated with reference to a rolling window of 4, 8, 16, 32, and 52 weeks, thus ensuring that we use only past values of the series to determine *m*.

### 2.3. Dataset

In this work, we analyze the price time series of four agri-commodities (aubergine, zucchini, green pepper, and cucumber) in the South region of Spain. We have focused on these four products because they are representative of the European vegetables market, as they represent a significant percentage of the total imports and exports.

The units of the prices are measured in EUR per kilogram. Each time series consists of daily prices collected over five years, and we must note that a typical week consists of six observations, since the market is closed on Sundays.

**Figure 1:**
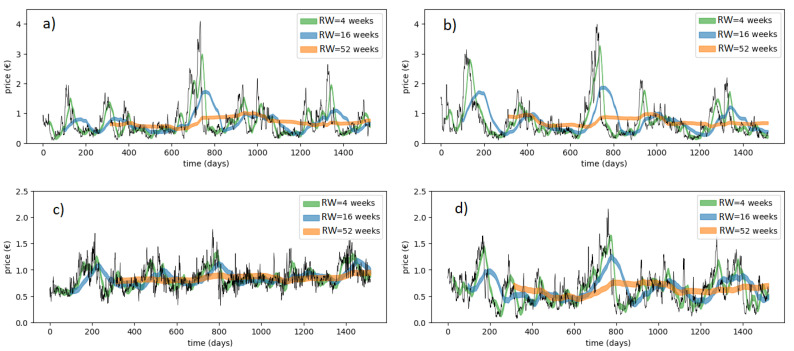
The figure shows the evolution of the price and its corresponding moving average for different rolling windows RW for (**a**) aubergine, (**b**) zucchini, (**c**) pepper, and (**d**) cucumber.

Figure 1 shows the evolution of the price of each commodity during the period 2015–2019. As it can be seen, all products present high volatility and despite showing a seasonal component, the noise component is still very important. In addition, we have included in the figure the moving averages for different rolling windows.

In Table 1, the Hurst exponent, average price, and standard deviation of each time series are shown. All of the products present a global Hurst below 0.45, i.e., antipersistent. Green pepper is the most antipersistent time series (H=0.18), and also the commodity with the highest and least volatile price.

## 3. Results

The main goal of our paper is to analyze if H can help to anticipate the future trend of the price of four different agri-commodities, and discuss the difference in performance when considering different rolling windows (RW). To this end, we will analyze whether the probability of MR in the short term is more likely during anti-persistent days than on persistent days or not.

### 3.1. Evolution of the Hurst Exponent

**Figure 2:**
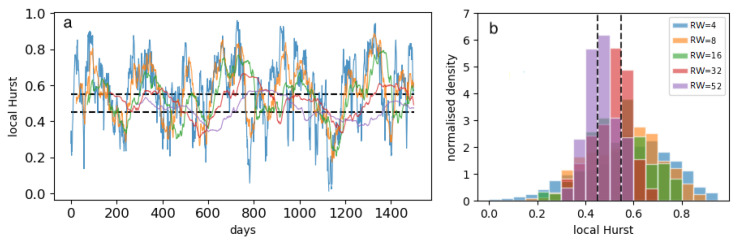
(**a**) The evolution of the local Hurst exponent for aubergine and different rolling windows. (**b**) Histogram of aubergine local Hurst exponent values. In both panels the dashed lines reflect the selected thresholds of 0.45 and 0.55.

To achieve this goal we begin by computing H as described in the methods section, over our time series for different values of RW (4, 8, 16, 32, and 52 weeks). Thus, for each day of our series we have computed the value of H for the mentioned windows. The evolution of H, for aubergine over time and its distribution, can be found in Figure 2. Panel B of the figure shows the distribution of H, which approximately follows a normal distribution. The mean value of H depends on the commodity and RW, but for most cases (except for green pepper) is close to 0.5. The exact values for each combination can be found in Table 2 and Table 3.

We found that for the four products, H varies over time, alternating periods of persistence, anti-persistency, and neutral regimens. Changes over days tend to be relatively smooth, and when the series enters one of the three regimens it keeps for a while. This effect is illustrated in panel B of Figure 2, which shows the case of aubergine.

### 3.2. Mean Reversion

**Figure 3:**
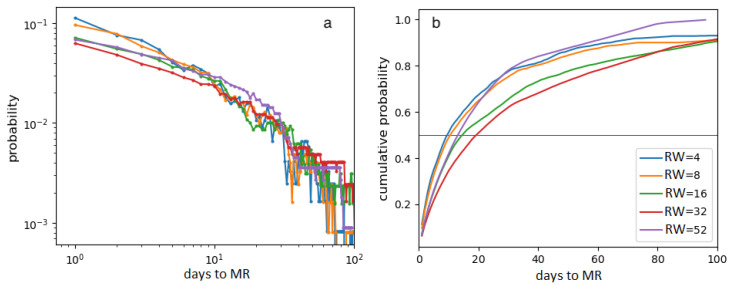
(**a**) Probability mass distribution of days to MR for the aubergine time series and for various RW. (**b**) The corresponding cumulative probability distributions.

In the second step, we compute for each day and RW the days to MR—this is the number of days left before the price will return or cross the mean. The days to MR follow a heterogeneous distribution, where for all the RWs over 50% of the observations revert to the mean in less than 20 days, while a small fraction take over 100 days. This can be observed in Figure 3a), which shows the probability mass functions (PMF) of each RW for aubergine.

When exploring the relation between H and days to MR, we find that the product (pepper) with a significantly smaller value of H, and in an anti-persistent regime (0.22–0.32), generally reverts to the mean more rapidly. For example, when conducting the analysis with RW=16 weeks, the price of pepper returns to the mean in an average of 5 days, while for the other products this value ranges between 11 and 22 days.

**Figure 4:**
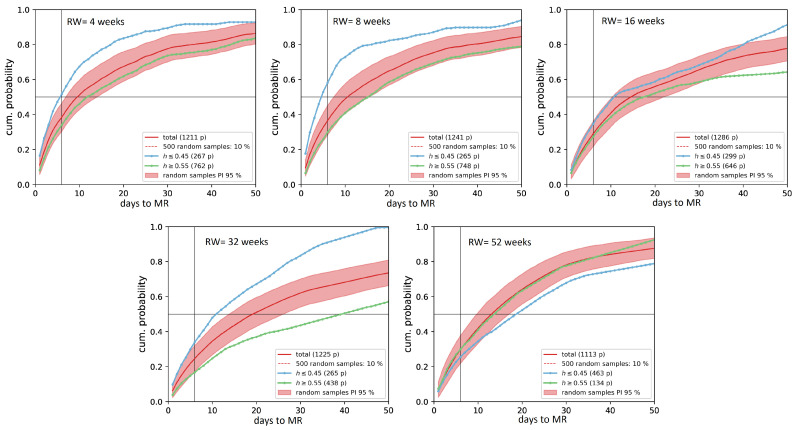
Cumulative probability of days to MR for the price time series of aubergine for different rolling windows RW. The blue curve represents the antipersistent regime, while the green curve represents the persistent regime. The horizontal line shows the 50% of probability, and the vertical one marks 6 days. The figure also shows the 95% level of confidence for the 500 random sub-samples.

Next, we analyze if the probabilities that the price will revert to the mean are significantly different for persistent and anti-persistent periods for each of the rolling windows. To this end, we classify each observation of the time series into persistent (H≥0.55), neutral (0.45<H<0.55), and anti-persistent (h≤0.45). Thus, we classify each day into one of the three different groups. We calculate the cumulative probability distribution of the days to MR for the persistent group, the anti-persistent group, and the total population and compare them for all the RWs. Figure 4 shows these distributions for aubergine.

We find that for all the RWs, except for 52 weeks, the cumulative distribution of days to MR significantly differs for the two regimens, the probability of MR being higher for anti-persistent days. In agreement, with this observation, the curve for the total population lies in-between both. Thus, anti-persistent days revert to the mean more quickly than persistent ones, which at the same time revert with lower probability than the global population. For the mentioned RW of 52 weeks (1 year), we observe the contrary effect, where the probability of MR in *d* or less days is always smaller for the anti-persistent regimen, the persistent group and the total population exhibiting a very similar behavior.

To test whether the observed behavior for the persistent and antipersistent groups could be random, we take random samples of the total population and compare their behavior to both groups. We do so, because the persistent and antipersistent groups are subsamples of the total population. Thus, there is a chance that by choosing a random subsample of similar size we find a similar effect. In such cases the effect we have observed would not be significant. Thus, we have randomly selected 500 subsamples and computed the cumulative distribution of days to MR for each one of them. In Figure 4, we have included the 2.5% and 97.5% percentiles so that they can be compared with the curves of the two groups. We find that for all the RWs, except 32 weeks, the effect of the persistent group is not likely to be significant as the curves lie in-between the two percentiles. However, for RW=32 weeks the probability of MR in 10 or less days for persistent days is below 30% and for 20 days it is below 40%. This means that we are around 70% confident that the price will stay at the current side of the border of the mean during the following 10 days, and 60% that it will also stay in the next 20 days. Hence, when computed with a RW of 32 weeks, H≥0.55 seems to be a good and informative indicator to anticipate the range in which the price will move in the medium-long term.

On the other hand, the anti-persistent group differs from the total population and the random samples for RWs of 4, 8, and 32 weeks, but not for 16 weeks. When analyzing our data, we can see that the most informative RWs are the shorter ones: 4 and 8, as the gap between the antipersistent group and the total population is the largest. This effect is especially relevant in the case of 8 weeks, where the probability of MR in one week is 58%, while the value for the total population is under 40%. Thus, an indicator detecting points where H≤0.45 would be informative for movements in the short term, as we will have 58% of probabilities that the price will return to its average. Hence, if the actual price is below the average we can anticipate an uptrend, and if the price is below we can expect a drop in the prices for the following week.

### 3.3. Paired t-Test

To further test the effect of persistence and anti-persistence in the expected days to MR, we adopt a quasi-experimental design, through which we can compare persistent and antipersistent observations against a control group. To this end, we perform two dependent *t*-tests for paired samples. The first one is to measure the effect of persistence and the second one is to measure the effect of anti-persistence. We use a dependent paired t-test, because our observations are extracted from a time series of prices, and thus can not be considered independent. In particular, we control for the commodity and the week, since the time series of prices have a marked seasonal component. Thus, for the persistence experiment we match the number of days to MR of each persistent observation to the number of days to MR on a randomly selected observation of the same week and commodity in the control group (all non antiperspirant observations). The anti-persistence experiment is designed analogously.

**Figure 5:**
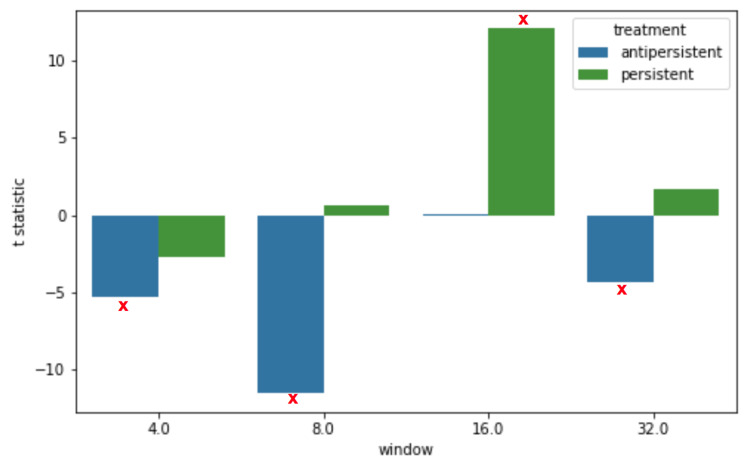
Results of the paired t-test for the persistence (H≥0.55) and antipersistence (H≤0.45) experiments. The figure shows the t-statistic, measured in days, for both experiments and the different RWs (RW = 4, 8, 16, 32 weeks). The significant differences have been marked with a red cross (*p*-value < 0.01).

The results of both tests for the RWs of 4, 8, 16, and 32 weeks are summarized in Figure 5. As it can be seen for all the RWs except for RW = 16, the MR occurs significantly faster for antipersistent observations. The most informative RW is the one of 8 weeks, where for antipersistent days we can expect MR to happen on average 12 days faster. In contrast, the effect of persistence is not so evident in our data, and we only find a significant effect for RW = 16, where in persistent periods MR takes on average 12 days longer. Note that we have not included the RW = 52 case, as there were not enough paired observations for the results to be reliable.

## 4. Discussion

In this paper we contribute to understand the agri-food market by exploring whether the market presents long term memory properties or not for several agri-commodities. To this end, we have computed the local Hurst exponent for several values of RW and measured the relation between its evolution and the probability that the price will revert to the mean. We found that in general for antipersistent days MR is more probable in the short term and occurs faster than for persistent and neutral days. The only value of RW for which we could not find a significant effect was RW=16, while the most informative one was RW=8.

The fact that MR is more probable and happens faster during antipersistent periods means that H can be a good indicator to anticipate the future motion and help actors operating in this market, such as co-ops or supermarkets. More particularly, it is important to discuss its implications to anticipate the price and operate in this market in the short-term. For RW=8 weeks we found that for antipersistent days almost 60% of the times MR will happen in one week or less, a magnitude significantly larger than what is expected for the full population, where the chances of MR in one week are below 45%. This fact, shows that H can be helpful to operate in such a market as it provides information to anticipate the future trend of the price. If the price is below the average, the operator will know that there is a high chance that the price will go up, while when the price is above the average a downtrend is very probable. We focus on the one week resolution, because anticipating MR in the long term is not so useful in this market. For example, knowing that MR will happen during the following two months, but not knowing when, means that the operator has to trade daily tons of fresh products with a high uncertainty on when the price movement will happen.

On the other hand, market indicators related to persistence can be related to the fact that MR is not very probable or will happen slowly. Thus, this kind of indicator is more useful to operate in the market in the long term. The fact that there is a low probability of MR for the following days is not too informative, as price time series present autocorrelation and the price from one day to another usually does not present big differences. In contrast, knowing that MR is not probable in the long term will help to anticipate the range in which the price will oscillate in the following months. When the price is above the average, knowing MR is not probable, and it is useful to know the lower barrier that the price will not cross in the following weeks and months. Likewise, when the price is below the average we have an upper frontier that the price is unlikely to cross. Thus, this information helps the actors of the market to negotiate long term contracts, which is very common between supermarkets and co-ops, where the second commits to provide a minimum quantity of tons during the following months to the second one for a fixed price.

A relevant research topic that we plan to explore in future work is the development of a methodology to find the optimal rolling window to use when computing the local Hurst exponent for each price time series. Thus, we aim to analyze a wider variety of products that follow different dynamics, and find their corresponding best rolling window.

## Figures and Tables

**Table 1:** Total Hurst, H, and mean and standard deviation of price for the four time series shown in Figure 1.

	Aubergine	Zucchini	Green Pepper	Cucumber
*H*	0.36	0.40	0.18	0.32
$\bar{p}\pm \sigma_{p}$	0.70±0.53	0.75±0.63	0.85±0.22	0.64±0.33
$p_{\max}-p_{\min}$	3.99	3.89	1.45	2.08

**Table 2:** This table shows the median of days to MR and mean local Hurst ($\bar{H}$) for the studied rolling windows *RW* =4,8,15,32,52 weeks for aubergine and zucchini. The global value of H of each product is also shown in the top row.

	Aubergine (H=0.36)		Zucchini (H=0.40)	
RW	**Days to MR**	$\bar{H}\pm \sigma$ _***H***_	**Days to MR**	$\bar{H}\pm \sigma$ _***H***_
4	10	0.57 ± 0.18	10	0.57±0.20
8	11	0.56±0.14	12	0.57±0.15
16	15	0.54±0.12	22	0.55±0.12
32	20	0.58±0.08	24	0.52±0.10
52	14	0.46±0.06	17	0.49±0.07

**Table 3:** This table shows the median of days to MR and mean local Hurst ($\bar{H}$) for the studied rolling windows RW=4,8,16,32,52 weeks for green pepper and cucumber. The global Hurst of each product is also shown in the top row.

	Green Pepper (H=0.18)		Cucumber (H=0.32)	
RW	**Days to MR**	$\bar{H}\pm \sigma$ _***H***_	**Days to MR**	$\bar{H}\pm \sigma$ _***H***_
4	3	0.32±0.16	11	0.52±0.19
8	4	0.28±0.10	11	0.53±0.12
16	5	0.26±0.07	14	0.50±0.07
32	5	0.25±0.05	15	0.46±0.05
52	4	0.22±0.04	14	0.41±0.06

## Data Availability

The datasets used for this study are publicly available from Observatorio de Precios y Mercados, Junta Andalucia, Spain at https://www.juntadeandalucia.es/agriculturaypesca/observatorio/servlet/FrontController?action=Static&url=introduccion.jsp and agroprecios.com at https://www.agroprecios.com/es/precios-subasta/.
